# The association between hypomagnesemia and poor glycaemic control in type 1 diabetes is limited to insulin resistant individuals

**DOI:** 10.1038/s41598-022-10436-0

**Published:** 2022-04-19

**Authors:** Lynette J. Oost, Julia I. P. van Heck, Cees J. Tack, Jeroen H. F. de Baaij

**Affiliations:** 1 Department of Physiology, Radboud Institute for Molecular Life Sciences, Radboud University Medical Center, P.O. Box 9101, 6500 HB Nijmegen, the Netherlands; 2 Department of Internal Medicine, Radboud Institute for Molecular Life Sciences, Radboud University Medical Center, Nijmegen, 6500 HB the Netherlands

**Keywords:** Endocrine system and metabolic diseases, Kidney

## Abstract

In a cohort of adults with type 1 diabetes, we examined the prevalence of hypomagnesemia and the correlation of serum magnesium levels with metabolic determinants, such as glycaemic control (as HbA_1c_), inflammatory markers and circulating cytokines. Furthermore, we assessed if a surrogate for insulin resistance is essential for the possible association of serum magnesium with metabolic determinants. Individuals with type 1 diabetes, aged above 18 years, were included and clinical characteristics were obtained from questionnaires and clinical records. In venous blood samples we measured cytokines and adipose-tissue specific secretion proteins. Serum magnesium concentrations were measured and correlated with clinical data and laboratory measurements using univariate and multivariate regression models. Hierarchical multiple regression of serum magnesium with insulin resistance was adjusted for diabetes and potential magnesium confounders. The prevalence of hypomagnesemia (serum magnesium levels < 0.7 mmol/L) was 2.9% in a cohort consisting of 241 individuals with type 1 diabetes. The magnesium concentration in the cohort was not associated with HbA_1c_ (*r* = − 0.12, *P*-value = 0.068) nor with any inflammatory marker or adipokine. However, insulin dose (IU/kg), a surrogate measure of resistance in type 1 diabetes, moderated the association of serum magnesium (mmol/L) with HbA_1c_ (mmol/mol) with a B coefficient of − 71.91 (95% CI: − 119.11; -24.71), *P*-value = 0.003) and Log_10_ high-sensitivity C-reactive protein (Log_10_ mg/L) − 2.09 (95% CI: − 3.70; − 0.48), *P*-value = 0.011). The association of low serum magnesium levels with glycaemic control (HbA_1c_) and high-sensitivity C-reactive protein in individuals with type 1 diabetes is limited to subjects using a high insulin dose and suggests that insulin resistance, a type 2 diabetes feature, is a prerequisite for hypomagnesemia.

## Introduction

A blood magnesium (Mg^2+^) concentration below 0.7 mmol/L is prevalent in people with type 2 diabetes in comparison to non-diabetic subjects, with reported percentages ranging from 13.5 to 47.7%^1^. Hypomagnesemia is associated with poor glycaemic control and progression from pre-diabetes to diabetes^2,3^, while some Mg^2+^ supplementation studies have shown to improve glycaemic control^4,5^. Moreover, maintaining physiological Mg^2+^ levels has been suggested to decrease the risk of developing cardiovascular diseases (CVD) in type 2 diabetes^6–8^.

Hypomagnesemia has also been reported in studies with type 1 diabetes individuals in the eighties and early nineties^9,10^. However, these results cannot be extrapolated to the present as treatment of type 1 diabetes has significantly improved over the past decades, resulting in better glycaemic control. Indeed, a recent study demonstrates that the prevalence of hypomagnesemia is only 4.3% in a cohort of type 1 diabetes^11^. Hence, novel studies studying the prevalence and identifying the factors that contribute to hypomagnesemia are warranted.

Here we assess the prevalence of hypomagnesemia in a cohort of type 1 diabetes adults and investigate whether Mg^2+^ levels are associated with clinical characteristics (sex, age, duration of diabetes, smoking and alcohol use), HbA_1c_, insulin sensitivity, body mass index (BMI), inflammatory markers and adipokines.

## Subjects and methods

### Subjects

Participants were selected from the outpatient diabetes clinic of the Radboud University Medical Center, the Netherlands. Inclusion criteria were a diagnosis of type 1 diabetes (based on clinical diagnosis) and age above eighteen years. Pregnant women were excluded.

This project is part of the Human Functional Genomics Project (HFGP)^12^. Ethical approval for the study was obtained from the Institutional Review Board of the Radboud University Medical Center (NL54214.091.15, 2015–1930 and NL42561.091.12, 2012–550). Participant inclusion and experiments were conducted according to the principles expressed in the Declaration of Helsinki. All participants gave written informed consent before participation.

### Demographic and laboratory analysis

Clinical characteristics, including age (years), sex (men/women), BMI (kg/m^2^), blood pressure (mmHg), smoking status (current, former, never), alcohol use (yes, no), duration of diabetes (years), HbA_1c_ (mmol/mol), insulin dose (units’ insulin per day), total cholesterol (mmol/L), triglycerides (TG) (mmol/L) and high density Lipoprotein (HDL) (mmol/L) were obtained from questionnaires and clinical records. Insulin resistance markers were calculated according to Bîcu et al.^13^. Venous blood was collected after an overnight fasting period. We measured high-sensitivity C-reactive protein (hs-CRP), IL-18 and IL-18 binding protein (IL-18BP), established inflammatory markers that are often elevated in individuals with diabetes, in the EDTA collected plasma samples (R&D duoset ELISA, MN, USA). Serum samples were measured for Mg^2+^ using a calibrated standardized colorimetric assay with a coefficient of variation of 1.98% (Cobas C8000; Roche Diagnostics, Risch-Rotkreuz, Switzerland).

### Statistical analysis

Data are presented as percentages, mean ± SD or median with interquartile range for skewed variables. Pearson’s correlation tests (continuous variables), Point-biserial correlations (dichotomous variables), or one-way ANOVA analysis (> 2 groups) were carried out to determine the correlation or difference between serum Mg^2+^ concentrations with demographic parameters or other laboratory measurements in total cohort and based on insulin treatment in units per kg body weight. Independent-samples t-test was run to assess differences between the mean of the high insulin dose (> 0.70 units per kg body weight, IU/kg)) versus low insulin dose (≤ 0.70 IU/kg).

Multivariate analysis was used to assess if serum Mg^2+^ has multiple interaction factors in the association with HbA_1c_, BMI, hs-CRP and Leptin. The linear relationship between continuous variables was assessed by scatterplot. Multicollinearity was assessed by Pearson correlations (*r* < 0.9). Hierarchical multiple regression was performed to identify if insulin dose (IU/kg) as continuous variable, significantly moderates the association of serum Mg^2+^ with HbA_1c_, BMI, hs-CRP and Leptin. Skewed dependent variables were Log_10_ transformed. Homoscedasticity was assessed by visual inspection of the studentized residuals plotted against the predicted values for high versus low insulin dose (IU/kg) individuals. The interaction model of serum Mg^2+^ (mmol/L) * insulin dose (IU/kg) as a continuous variable was adjusted for possible confounders: model 1 is age (years) and sex (men/women) adjusted, model 2 is adjusted for duration of diabetes (years), estimated glomerular filtration rate (eGFR) (< 60, 60–90, > 90 mL/min/1.73m^2^), alcohol use (yes, no), smoking (current, former, never), TG (mmol/L), LDL cholesterol (mmol/L), systolic blood pressure (mmHg), statins use (yes, no) and proton pump inhibitor (PPI) use (yes, no). Plots of the association of serum Mg^2+^ with outcome variables, crude and corrected for confounders, are visualized using the PROCESS macro in SPSS^14^. Missing data (< 13%) were imputed for regression analysis using Predictive Mean Matching combing ten iterations and thirteen imputation sets into one imputation model. All data analyses were performed using SPSS for Windows (v25.0.0.01, IBM). P-values ≤ 0.05 were considered statistically significant.

## Results

In total, 241 individuals with type 1 diabetes were included in the cohort. Demographic data and laboratory results are shown in Table 1. The mean serum Mg^2+^ concentration was 0.84 ± 0.10 mmol/L, with 7 people (2.9%) that had hypomagnesemia (serum Mg^2+^  < 0.7 mmol/L). Serum Mg^2+^ concentrations were correlated with duration of diabetes (*r* = 0.15, *P*-value = 0.017), serum creatinine (*r* = 0.14, *P*-value = 0.032) and eGFR (F (2238) = 3.73, *P*-value = 0.037). The association of serum Mg^2+^ with HbA_1c_ was (*r* = − 0.12, *P*-value = 0.068). All other variables such as demographic characteristics (sex, age, alcohol use, smoking) and laboratory measurements (cholesterol, inflammatory markers and adipokines) were not associated with serum Mg^2+^. Since studies in individuals with type 2 diabetes have shown strong negative correlations between serum Mg^2+^ and parameters related to insulin sensitivity^15,16^, we divided the cohort into quartiles based on insulin dose, (determined by the amount of insulin treatment in units per kg body weight). In the quartile with high insulin dose (> 0.70 IU/kg) but not in the other quartiles, a clear, inverse, correlation between Mg^2+^ serum level and HbA_1c_ (*r* = − 0.26, *P*-value = 0.047) was found. In this quartile, Mg^2+^ serum level also correlated with sex (men) (*r* = 0.30, *P*-value = 0.021), and inversely with BMI (*r* = − 0.29, *P*-value = 0.026), Log_10_ hs-CRP (*r* = − 0.39, *P*-value = 0.003) and Log_10_ Leptin (*r* = − 0.37, *P*-value = 0.004). The correlations of serum Mg^2+^ with HbA_1c_, in subjects with a low insulin dose (≤ 0.70 IU/kg), were (*r* = − 0.07, *P*-value = 0.362) for, BMI (*r* = 0.03, *P*-value = 0.714), Log_10_ hs-CRP (*r* = − 0.01, P-value = 0.900) and Log_10_ Leptin (*r* = 0.06, *P*-value = 0.464).

**Table 1:** Characteristics of individuals with type 1 diabetes in the DM300 cohort (*n* = 241).

**Demographic variables**	
Men (%)	54
Age (years)	52 ± 16
Duration of diabetes (years)	28.4 ± 15.7
Alcohol use, *n* (%)	73
**Smoking, *n* (%)**	
Current	11
Former	40
Never	48
**Metabolic variables**	
BMI (kg/m^2^)	25.8 ± 4.4
Hba_1c_ (%)	8.0 ± 1.3
HbA_1c_ (mmol/mol)	64 ± 15
Daily insulin dose (IU/kg)	0.57 (0.44–0.70)
Albumin Creatine ratio (mg/mmol)	0.80 (0.50–1.90)
Serum creatinine (µmol/L)	73 (64–78)
Total cholesterol (mmol/L)	4.65 ± 0.88
TG (mmol/L)	0.95 (0.72–1.38)
LDL (mmol/L)	2.65 ± 0.76
HDL (mmol/L)	1.67 ± 0.54
Systolic Blood Pressure (mmHg)	131 ± 17
Diastolic Blood Pressure (mmHg)	73 ± 10
**eGFR (mL/min/1.73m**^**2**^**), *n* %**	
< 60	13
60–90	31
> 90	56
**Cytokines, hormones and inflammatory markers**	
hs-CRP (mg/L)	0.90 (0.36–2.25)
IL18-bp/IL18 complex (ng/mL)	1.74 (1.30–2.12)
Alpha-1 antitrypsin (mg/mL)	0.46 (0.36–0.62)
Adiponectin (µg/mL)	4.12 (2.79–6.59)
Leptin (ng/mL)	7.85 (3.30–16.44)
Resistin (pg/mL)	12.66 (10.20–16.10)
**Medication use, *n* (%)**	
Metformin	2 (0.8)
PPI	34 (14.1)
Statins	84 (34.9)

Characteristics are presented as n (%), or mean ± SD, or median (interquartile rang). BMI = Body Mass Index, HDL = high density lipoprotein, hs-CRP = high-sensitivity C-reactive protein, IL18-bp = interleukin18-binding protein, PPI = proton pump inhibitor, TG = triglycerides.

To validate that the high insulin dose (> 0.70 IU/kg) (*n* = 61) group is truly insulin resistant we assessed widely used insulin resistance markers: TG / HDL ratio, TG levels and total cholesterol/HDL ratio^13^. All insulin resistance markers were statistically higher in the group that used > 0.70 IU/kg compared to the low insulin use group, with a difference of 0.64 (95% CI: 0.54; 0.86), *t*(205) = 6.14, *P*-value =  < 0.001 for TG / HDL ratio, 0.55 (95% CI: 0.33; 0.77), *t*(205) = 4.97, *P*-value =  < 0.001 for TG and 0.52 (95% CI: 0.24; 0.80), *t*(2.13) = 3.64, *p*-value =  < 0.001.

To identify if there was an interaction between serum Mg^2+^ with sex and insulin dose on outcome variables HbA_1c_, BMI, Log_10_ hs-CRP and Log_10_ Leptin we performed a multivariate analysis. The interaction effect between sex and serum Mg^2+^ on the combined dependent variables (HbA_1c_, BMI, Log_10_ hs-CRP and Log_10_ Leptin) was not statistically significant, F(4, 232) = 0.55, *P*-value = 0.703, partial *η*2 = 0.009. There was a statistically significant interaction effect between insulin dose and serum Mg^2+^ on the combined dependent variables, F(4, 232) = 2.97, *P*-value = 0.024, partial *η*2 = 0.059. The results of the multivariate analysis per individual dependent variable is reported in Supplementary table 1.

**Figure 1:**
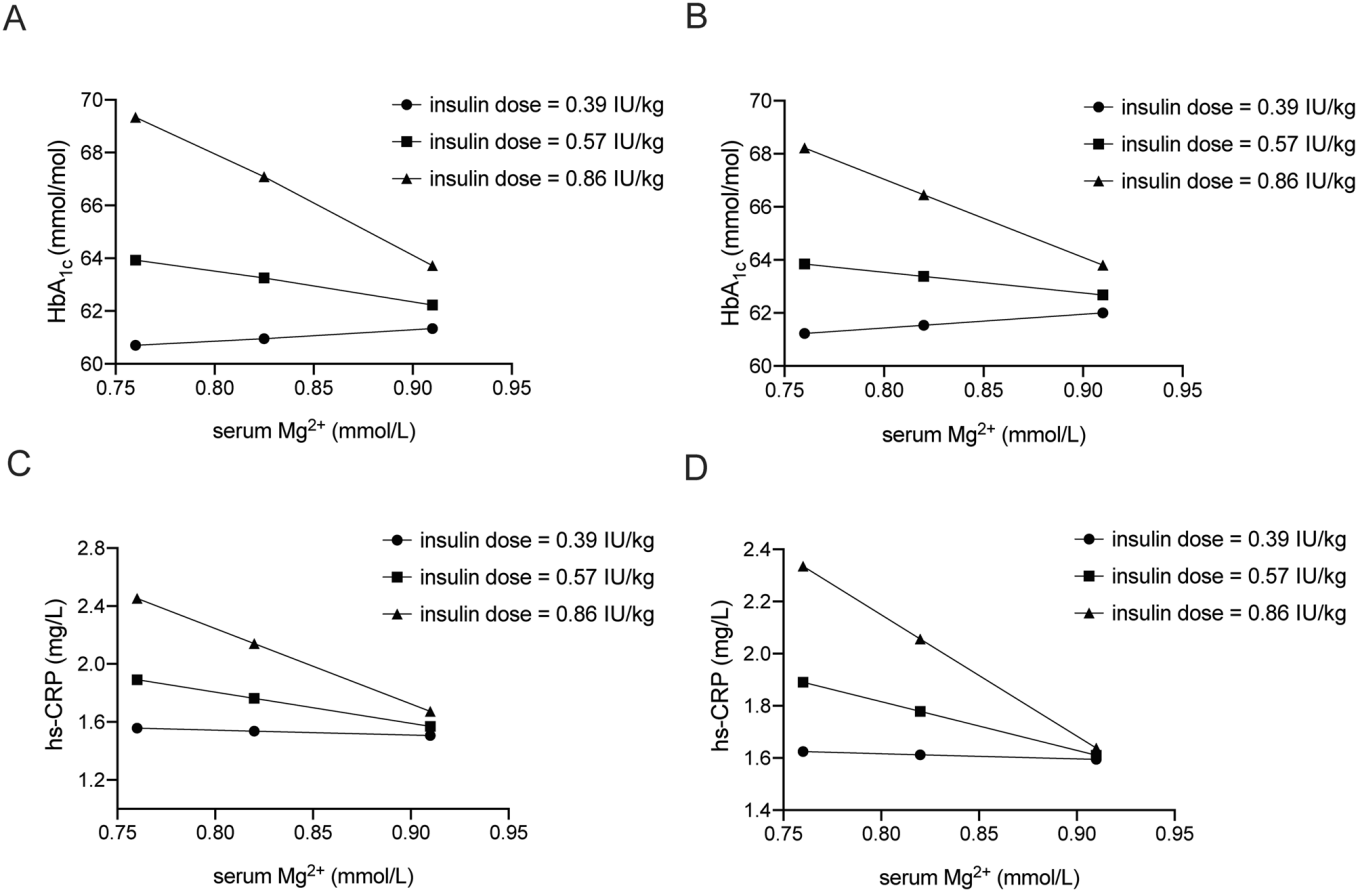
Plots of the association of serum Mg^2+^ with HbA_1c_ and hs-CRP categorized by insulin dose. (**A, C**) Crude models and (**B, D**) Model 2, adjusted for: age, sex adjusted, duration of diabetes (years), eGFR (< 60, 60–90, > 60 mL/min/1.73m^2^), alcohol use (yes/no), smoking (current, former, never), SBP (mmHg), TG (mmol/L), LDL cholesterol (mmol/L), statins (yes/no) and PPI (yes/no) drugs. eGFR = estimated glomerular filtration rate, HbA_1c_ = hemoglobine A1c, hs-CRP = high-sensitivity C-reactive protein, LDL = low-density lipoprotein, Mg^2+^ = magnesium, PPI = proton pump inhibitor, SBP = systolic blood pressure, TG = triglycerides.

As follow-up analysis, hierarchical multiple regression was used to assess the interaction effect of continuous insulin resistance with serum Mg^2+^ and analyzed by multivariable linear regression on the association of outcome variables: HbA_1c_, BMI, Log_10_ hs-CRP and Log_10_ Leptin. Insulin dose moderated the effect of serum Mg^2+^ on HbA_1c_ and Log_10_ hs-CRP, as evidenced by a statistically significant increase in total variation explained of 5.0% (F(1, 237) = 13.71, *P*-value =  < 0.001) for HbA_1c_ and 3.5% (F(1, 237) = 8.82, *P*-value = 0.004) for Log_10_ hs-CRP. After adjusting for confounders, the moderator effect of insulin dose and serum Mg^2+^ remained in the association with HbA_1c_ − 71.91 (95% CI: − 119.11; − 24.71), *P*-value = 0.003) (Table 2) and Log_10_ hs-CRP − 2.09 (95% CI: − 3.70; − 0.48), *P*-value = 0.011 (Table 3). Plotting the interaction shows that insulin dose is critical for the negative association of serum Mg^2+^ with HbA_1c_ and hs-CRP (Fig. 1). The crude associations between serum Mg^2+^, HbA_1c_, Log_10_ hs-CRP and insulin dose are visualized in Supplementary Fig. 1. The interaction effect of insulin resistance and serum Mg^2+^ with obesity markers BMI and Log_10_ Leptin were not significant after adjusting for confounders (Supplementary table 2 and 3).

**Table 2:** Interaction effect of the moderator insulin dose and serum Mg^2+^ associated with HbA_1c_.

	Crude model		Model 1		Model 2	
	B (95% CI)	*P*-value	B (95% CI)	*P*-value	B (95% CI)	*P*-value
Constant	24.95 (− 4.21; 54.10)	0.094	26.90 (− 2.38; 56.18)	0.072	24.72 (− 11.04; 60.47)	0.175
Serum Mg^2+^ (mmol/L)	37.88 (3.97; 71.80)	0.029	32.78 (− 0.84; 66.41)	0.056	32.66 (− 1.76; 67.08)	0.063
Insulin dose (IU/kg)	84.12 (43.82; 124.42)	< 0.001	76.25 (36.12; 116.38)	< 0.001	69.25 (28.43; 110.06)	0.001
Serum Mg^2+^ (mmol/L) * insulin dose (IU/kg)	− 86.97 (− 133.72; -40.22)	< 0.001	− 77.08 (− 123.73; -30.43)	0.001	− 71.91 (− 119.11; -24.71)	0.003

Model 1 is age- and sex adjusted. Model 2 is adjusted for duration of diabetes (years), eGFR (< 60, 60–90, > 60 mL/min/1.73m^2^), alcohol use (yes/no), smoking (current, former, never), SBP (mmHg), TG (mmol/L), LDL cholesterol (mmol/L), statins (yes/no) and PPI (yes/no) drugs. eGFR = estimated glomerular filtration rate, LDL = low-density lipoprotein, Mg^2+^ = magnesium, PPI = proton pump inhibitor, SBP = systolic blood pressure, TG = triglycerides.

**Table 3:** Interaction effect of the moderator insulin dose and serum Mg^2+^ associated with Log_10_ hs-CRP.

	Crude model		Model 1		Model 2	
	B (95% CI)	*P*-value	B (95% CI)	*P*-value	B (95% CI)	*P*-value
Constant	− 1.04 (− 2.07; -0.004)	0.049	− 0.98 (-2.03; 0.06)	0.066	− 1.10 (− 2.34; 0.14)	0.081
Serum Mg^2+^ (mmol/L)	1.07 (− 0.14; 2.27)	0.082	0.91 (− 0.29; 2.11)	0.135	0.80 (− 0.038; 1.98)	0.182
Insulin dose (IU/kg)	2.25 (0.83; 3.67)	0.002	2.01 (0.58; 3.44)	0.006	1.88 (0.49; 3.27)	0.008
Serum Mg^2+^ (mmol/L) * insulin dose (IU/kg)	− 2.47 (− 4.13; 0.82)	0.003	− 2.17 (− 3.83; -0.52)	0.010	− 2.09 (− 3.70; − 0.48)	0.011

Model 1 is age- and sex adjusted. Model 2 is adjusted for duration of diabetes (years), eGFR (< 60, 60–90, > 60 mL/min/1.73m^2^), alcohol use (yes/no), smoking (current, former, never), SBP (mmHg), TG (mmol/L), LDL cholesterol (mmol/L), statins (yes/no) and PPI (yes/no) drugs. eGFR = estimated glomerular filtration rate, LDL = low-density lipoprotein, Mg^2+^ = magnesium, PPI = proton pump inhibitor, SBP = systolic blood pressure, TG = triglycerides.

## Discussion

This study shows that the prevalence of hypomagnesemia (Mg^2+^ blood levels < 0.7 mmol/L) in a contemporary cohort of type 1 diabetes adults is only 2.9%, which is comparable to the normal population^17^. We also demonstrate an inverse correlation of serum Mg^2+^ with glycaemic control (as HbA_1c_) and hs-CRP that seems to be dependent on insulin resistance.

The prevalence of hypomagnesemia in type 1 diabetes in the present cohort is significantly lower than in type 2 diabetes adults (13.5–47.7%) and historic cohorts of insulin-treated outpatients with diabetes^1,10,18^. Our study thereby confirms the low prevalence of 4.3% in a type 1 diabetes cohort of 207 patients published earlier this year^11^. In 1989, McNair et al*.* reported a high prevalence of hypomagnesemia (38%) and inverse correlation of serum Mg^2+^ with glucose levels^10^, but since then, diabetes care has substantially improved. The proportion type 1 diabetes individuals with good glycaemic control (HbA_1c_ from < 7.5%) has risen from 25 to 45% while the proportion of people with poor glycaemic control decreased from 40 to 16%^19^. Indeed, the average HbA_1c_ in our cohort (8.0%) was lower compared to earlier studies^9,20^, and comparable to the most recent study having a baseline HbA_1c_ of 7.6%^11^.

Older studies in type 1 diabetes cohorts have reported the negative association of Mg^2+^ blood levels with fasting glucose or HbA_1c_^20,21^. A recent study from Dijk et al*.* supports that there is no association with HbA_1c_ or obesity markers in a total cohort of people with type 1 diabetes. The study of Dijk et al*.* did not assess the effect of insulin resistance and also 86% the data regarding insulin dose was missing^11^. In our study, we show that serum Mg^2+^ is negatively associated with HbA_1c_ and Log_10_ hs-CRP in people that are probably insulin resistant. In a cohort with type 1 and 2 diabetes, the likelihood of having high CRP concentration increased with HbA_1c_ levels^22^. The CRP median in our study is comparable to levels measured in previous type 1 diabetes studies and on average still lower than in individuals with type 2 diabetes^23^. Interestingly, CRP is associated with a higher risk of developing type 2 diabetes^24^, while Mg^2+^ reduces type 2 diabetes incidence^25,26^. In the general population, low Mg^2+^ levels are associated with raised CRP concentration^27^, and oral Mg^2+^ supplementation reduces serum CRP levels^28^. Our results suggest that insulin resistance might be an important determinant in the relation of serum Mg^2+^ with glycaemic control. We did correct for confounders such as TG and SBP, because these are known to be positively correlated with metabolic insulin resistance. Adjusting for TG and SBP as confounders did not attenuate the moderation effect of insulin dose on the association of serum Mg^2+^ with HbA_1c_ or log transformed hs-CRP^29,30^. This suggests that there could be other factors than insulin resistance involved that contribute to hypertriglyceridemia and hypertension in people with type 1 diabetes^31,32^.

The results do explain the high prevalence of hypomagnesemia in type 2 diabetes with insulin resistance being the hallmark of this disorder. Insulin resistance is often associated with being overweight or obese, a factor that is becoming more common in type 1 diabetes too, resulting in the development of “double diabetes”^33^. The inflammatory marker CRP is even considered as a predictor for pre-diabetes, diabetes and fatty liver disease^34–36^. This suggests that a similar mechanism of hypomagnesaemia might occur in pathologies that are closely-related to the type 2 diabetes phenotype, such as pre-diabetes and fatty liver disease.

The strengths of this study includes the fact that we studied a large cohort over a wide age range, while other type 1 diabetes studies have determined the incidence in rather small sample sizes of children and adolescents^18,37^. Another advantage is that we have determined adipose tissue specific lipids and inflammatory cytokines. A limitation is the cross-sectional design study, although we do provide some insight in the mechanism of Mg^2+^ by using an explanatory statistical model.

In summary, this study shows that serum Mg^2+^ levels are negatively associated with glycaemic control and to inflammation (log_10_ hs-CRP), but this relationship is limited to people with type 1 diabetes who are probably insulin resistant. These results suggest that hypomagnesemia is not caused by diabetes per se, but that insulin resistance is the main determinant in the association of Mg^2+^ and glycaemic control in individuals with type 1 and type 2 diabetes.

## Supplementary Information

Supplementary Information.

## Data Availability

Existing ethical permits do not allow that personal data from this study are deposited in the public domain. The full dataset is available for researchers who meet the criteria for confidential data access as stipulated by participant informed consent and the Institutional Review Board of the Radboud University Medical Center (NL54214.091.15, 2015–1930 and NL42561.091.12, 2012–550).
