# Supplementary material for: The association between hypomagnesemia and poor glycaemic control in type 1 diabetes is limited to insulin resistant individuals
Source: Sci Rep. 2022 Apr 19;12:6433. doi: 10.1038/s41598-022-10436-0 (PMC9018833; doi:10.1038/s41598-022-10436-0)
Supplement: Supplementary file 1 — Supplementary Information. [file 41598_2022_10436_MOESM1_ESM.docx]

**The association between hypomagnesemia and poor glycaemic control in type 1 diabetes is limited to insulin resistant individuals**

**Scientific Reports**

**Online Supplementary Material**

Lynette J. Oost^1^, Julia I.P. van Heck^2^, Cees J. Tack^2^, Jeroen H.F. de Baaij^1^

^1^Department of Physiology, Radboud Institute for Molecular Life Sciences, Radboud university medical center, 6500 HB Nijmegen, the Netherlands

^2^Department of Internal Medicine, Radboud Institute for Molecular Life Sciences, Radboud university medical center, Nijmegen, 6500 HB, the Netherlands

Corresponding author:

Dr. Jeroen H.F. de Baaij, Department of Physiology, Radboud Institute for Molecular Life Sciences, Radboud university medical center, P.O. Box 9101, 6500HB, Nijmegen, The Netherlands. Phone: (+31) 24 3617347, E-mail: jeroen.debaaij@radboudumc.nl

Supplementary table 1. Multivariate analysis showing the interaction effect of insulin dose with serum Mg^2+^ and sex with metabolic outcome variables HbA_1c_, BMI, Log_10_ hs-CRP and Log_10_ Leptin.

| **Source** | **Dependent variables** | **Sum of squares** | **df** | **Mean square** | **F** | **P** |
| --- | --- | --- | --- | --- | --- | --- |
| Serum Mg^2+^ | HbA_1c_ | 1112.0 | 1 | 1112.0 | 5.751 | 0.019 |
|  | BMI | 70.8 | 1 | 70.8 | 3.817 | 0.055 |
|  | Log_10_ Leptin | 0.1 | 1 | 0.1 | 0.961 | 0.332 |
|  | Log_10_ hs-CRP | 1.6 | 1 | 1.6 | 7.156 | 0.009 |
| High insulin dose^1^ | HbA_1c_ | 905.1 | 1 | 905.1 | 4.681 | 0.045 |
|  | BMI | 101.8 | 1 | 101.8 | 5.491 | 0.021 |
|  | Log_10_ Leptin | 0.7 | 1 | 0.7 | 5.085 | 0.026 |
|  | Log_10_ hs-CRP | 1.9 | 1 | 1.9 | 8.485 | 0.005 |
| Sex | HbA_1c_ | 340.3 | 1 | 340.3 | 1.758 | 0.188 |
|  | BMI | 15.8 | 1 | 15.8 | 0.853 | 0.385 |
|  | Log_10_ Leptin | 0.4 | 1 | 0.4 | 2.983 | 0.086 |
|  | Log_10_ hs-CRP | 0.4 | 1 | 0.4 | 1.576 | 0.212 |
| Serum Mg^2+^ * high insulin dose^1^ | HbA_1c_ | 594.1 | 1 | 594.1 | 3.073 | 0.086 |
|  | BMI | 97.9 | 1 | 97.9 | 5.280 | 0.024 |
|  | Log_10_ Leptin | 0.7 | 1 | 0.7 | 4.381 | 0.039 |
|  | Log_10_ hs-CRP | 1.6 | 1 | 1.6 | 6.871 | 0.011 |
| Serum Mg^2+^ * sex | HbA_1c_ | 193.0 | 1 | 193.0 | 0.997 | 0.321 |
|  | BMI | 11.4 | 1 | 11.4 | 0.615 | 0.468 |
|  | Log_10_ Leptin | 0.03 | 1 | 0.03 | 0.211 | 0.648 |
|  | Log_10_ hs-CRP | 0.2 | 1 | 0.2 | 0.958 | 0.331 |
| Error | HbA_1c_ | 45474.7 | 235 | 193.5 |  |  |
|  | BMI | 4358.8 | 235 | 18.5 |  |  |
|  | Log_10_ Leptin | 35.3 | 235 | 0.2 |  |  |
|  | Log_10_ hs-CRP | 53.9 | 235 | 0.2 |  |  |
| Total | HbA_1c_ | 1036753.8 | 241 |  |  |  |
|  | BMI | 160525.5 | 241 |  |  |  |
|  | Log_10_ Leptin | 241.3 | 241 |  |  |  |
|  | Log_10_ hs-CRP | 59.8 | 241 |  |  |  |

^1^High insulin dose as a dichotomous variable with a cut-off of >0.70 IU/kg body weight. HbA_1c_= hemoglobine A_1c_, hs-CRP= high-sensitivity C-reactive protein, Mg^2+^= magnesium. HbA_1c_ in mmol/mol. BMI in kg/m^2^. Serum Mg^2+^ in mmol/L.

Supplementary table 2. Interaction effect of the moderator insulin dose and serum Mg^2+^ associated with BMI.

|  | **Crude model** | | **Model 1** | | **Model 2** | |
| --- | --- | --- | --- | --- | --- | --- |
|  | **B (95% CI)** | **P-value** | **B (95% CI)** | **P-value** | **B (95% CI)** | **P-value** |
| Constant | 17.60 (8.23; 26.96) | <0.001 | 16.70 (7.34; 26.05) | <0.001 | 14.46 (3.15; 25.77) | 0.012 |
| Serum Mg^2+^ (mmol/L) | 8.79  (-2.14; 19.72) | 0.115 | 7.27  (-3.55; 18.09) | 0.188 | 4.15  (-6.88; 15.19) | 0.460 |
| Insulin dose (IU/kg) | 15.98  (3.29; 28.67) | 0.014 | 14.36  (1.76; 26.96) | 0.026 | 11.89  (-0.89; 24.67) | 0.068 |
| Serum Mg^2+^ (mmol/L) * insulin dose (IU/kg) | -17.52  (-32.27; -2.76) | 0.020 | -15.42  (-30.10; -0.74) | 0.040 | -13.31  (-28.14; 1.52) | 0.078 |

Model 1 is age- and sex adjusted. Model 2 is adjusted for duration of diabetes (years), eGFR (<60, 60-90, >60 mL/min/1.73m^2^), alcohol use (yes/no), smoking (current, former, never), SBP (mmHg), TG (mmol/L), LDL cholesterol (mmol/L), statins (yes/no) and PPI (yes/no) drugs. eGFR= estimated glomerular filtration rate, LDL= low-density lipoprotein, Mg^2+^= magnesium, PPI= proton pump inhibitor, SBP= systolic blood pressure, TG= triglycerides.

Supplementary table 3. Interaction effect of the moderator insulin dose and serum Mg^2+^ associated with Log_10_ Leptin.

|  | **Crude model** | | **Model 1** | | **Model 2** | |
| --- | --- | --- | --- | --- | --- | --- |
|  | **B (95% CI)** | **P-value** | **B (95% CI)** | **P-value** | **B (95% CI)** | **P-value** |
| Constant | -0.26 (-1.25; 0.73) | 0.607 | 0.21 (-0.61; 1.04) | 0.504 | 0.42 (-0.57; 1.42) | 0.407 |
| Serum Mg^2+^ (mmol/L) | 1.31  (0.16; 2.47) | 0.026 | 0.79  (-0.15; 1.74) | 0.100 | 0.48  (-0.46; 1.42) | 0.317 |
| Insulin dose (IU/kg) | 2.03  (0.67; 3.40) | 0.004 | 1.10  (-0.02: 2.22) | 0.055 | 0.93  (-0.17; 2.03) | 0.096 |
| Serum Mg^2+^ (mmol/L) * insulin dose (IU/kg) | -2.35  (-3.94; -0.77) | 0.004 | -1.19  (-2.49; 0.12) | 0.074 | -1.04  (-2.31; 0.24) | 0.112 |

Model 1 is age- and sex adjusted. Model 2 is adjusted for duration of diabetes (years), eGFR (<60, 60-90, >60 mL/min/1.73m^2^), alcohol use (yes/no), smoking (current, former, never), SBP (mmHg), TG (mmol/L), LDL cholesterol (mmol/L), statins (yes/no) and PPI (yes/no) drugs. eGFR= estimated glomerular filtration rate, LDL= low-density lipoprotein, Mg^2+^= magnesium, PPI= proton pump inhibitor, SBP= systolic blood pressure, TG= triglycerides.


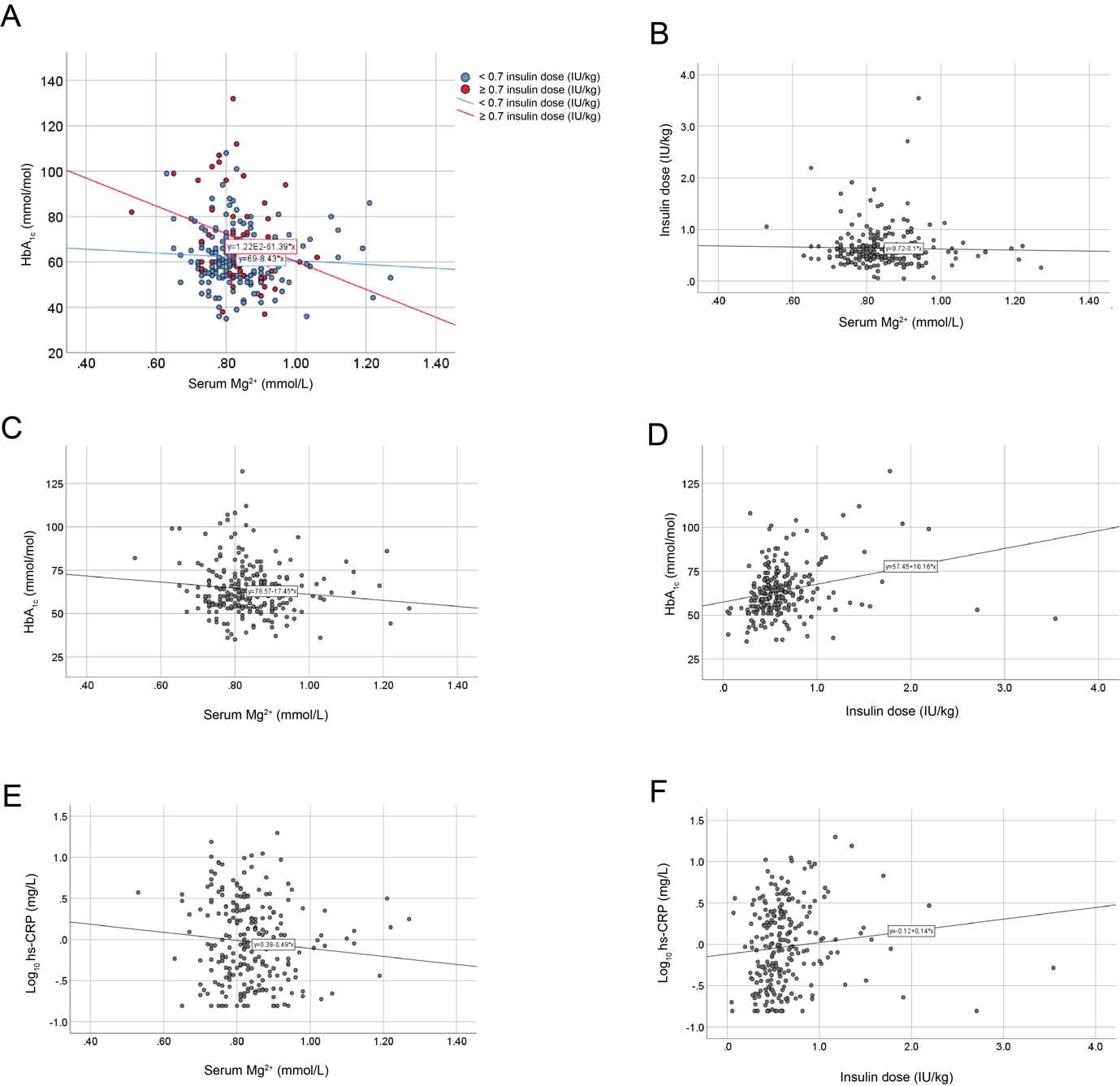


Supplementary figure 1. Crude data scatter plots of (A) serum Mg^2+^ (mmol/L) versus HbA_1c_ (mmol/mol) divided in subgroups based on insulin dose below and above 0.7 IU/kg, (B) serum Mg^2+^ (mmol/L) versus insulin dose (IU/kg), (C) serum Mg^2+^ (mmol/L) versus HbA_1c_ (mmol/mol), (D) insulin dose (IU/kg) dependence on HbA_1c_ (mmol/mol)_,_ (E) serum Mg^2+^ (mmol/L) versus Log_10_ hs-CRP (mg/L), and (F) insulin dose (IU/kg) versus Log_10_ hs-CRP (mg/L). Mg^2+^= magnesium, HbA_1c_= hemoglobine A_1c_, hs-CRP= high-sensitivity C-reactive protein.
